# The complete chloroplast genome of *Blumea axillaris* (Asteraceae: *Blumea*) and phylogenetic analysis

**DOI:** 10.1080/23802359.2026.2616120

**Published:** 2026-02-05

**Authors:** Ting-Ying Cao, Yan-Jie Wang, Yu-Lan Peng

**Affiliations:** a Sichuan Normal University, Chengdu, China; b Chengdu Institute of Biology, Chinese Academy of Sciences, Chengdu, China

**Keywords:** *Blumea axillaris*, chloroplast genome, phylogenetic analysis

## Abstract

*Blumea axillaris* is a medicinal herbaceous plant belonging to the genus *Blumea* in the Asteraceae family. This study reports the first complete chloroplast genome of *B. axillaris*, assembled as 151,043 bp with a typical quadripartite structure. It contains 134 genes (90 protein-coding, 36 tRNA, and 8 rRNA). A double inversion was detected relative to *Barnadesia caryophylla*. Phylogenetic analysis confirmed the sister relationship between *B. axillaris* and *B. oxyodonta*. These results provide valuable chloroplast resources for phylogenetic studies and resource development of *B. axillaris*

## Introduction

*Blumea axillaris* (Lam.) DC. (1825) is a herbaceous plant belonging to the genus *Blumea* in the Asteraceae family. The plant grows in fields and open grasslands from sea level to 2100 m in altitude (Pornpongrungrueng et al. 2016). The whole plant is used for symptoms such as pneumonia and coughing with wheezing (Jiang and Jiang 2016); in traditional Miao practices in China, the stems and leaves are pounded to make wine starter for enhancing aroma (Zhao et al. 2014).

The chloroplast genomes of angiosperms are typically circular molecules of 120–160 kb that adopt a quadripartite structure, wherein a large single-copy (LSC) region and a small single-copy (SSC) region are separated by two inverted repeat (IR) regions, and which collectively contain 110–130 unique genes (Daniell et al. 2016). The chloroplast genome, characterized by single-copy regions and conserved features, has become an ideal molecular marker due to its structural variations (such as inversions) and sequence polymorphisms. It is widely applied in the resolution of phylogeny at the genus level and in species identification (Shaw et al. 2007; Parks et al. 2009).

Previous studies have conducted preliminary phylogenetic investigations of the genus *Blumea* based on a limited number of molecular markers, such as the nuclear gene *ITS* and the chloroplast gene fragment *trnL-trnF* (Pornpongrungrueng et al. 2007, 2009; Chung et al. 2022). These studies indicated that taxa of the genus *Blumea* were resolved into three major clades: the *B. lacera* clade, the *B. densiflora* clade, and the *B. balsamifera* clade. Within the *B. lacera* clade, the works of Zhang et al. (2019) and Chung et al. (2022) supported the recognition of three subclades and two individual species. Specifically, *B. axillaris* was placed within subclade II of the *B. lacera* clade and formed a sister-group relationship with *B. oxyodonta*. However, characterizations and phylogenetic analyses based on the complete chloroplast genome of *B. axillaris* are still lacking.

Therefore, this study sequences, assembles, and annotates the complete chloroplast genome of *B. axillaris* to further clarify its phylogenetic relationships and provide resources for DNA-based identification.

## Materials and methods

**Figure 1.**
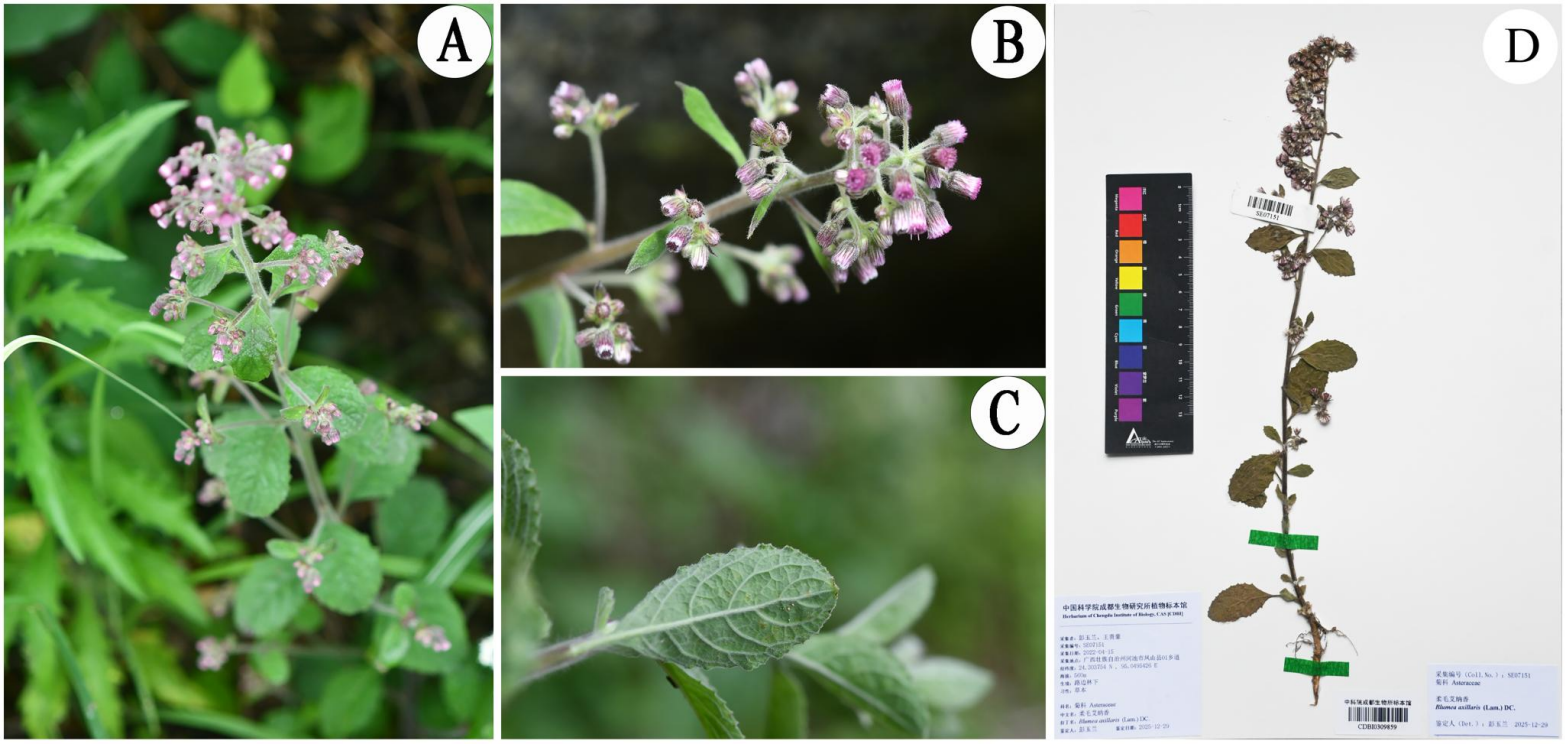
The photos of *Blumea axillaris*. (A) Whole plant, (B) flowering plant, (C) abaxial leaf surface, and (D) herbarium of *Blumea axillaris.* Photograph by Yulan Peng. Key morphological features: erect stems (20–80 cm) bear long pilose and sparse glandular hairs. Leaves are petiolate with elliptic-ovate, attenuate-based, pubescent-glandular blades. Flowers: purple to light red.

Fresh leaves of *B. axillaris* (Figure 1) were collected from Fengshan County, Hechi City, Guangxi Zhuang Autonomous Region, China (106°56′42.03ʺ E, 24°18′13.51ʺ N, elevation ca. 560 m) on 15 April 2022. The plant was identified by Yu-Lan Peng based on morphological characteristics. The voucher specimen has been deposited at the Herbarium of the Chengdu Institute of Biology (CDBI), Chinese Academy of Sciences (contact person: Yu-Lan Peng, email: pengyl@cib.ac.cn) under the voucher number SE07151 (Figure 1(D)).

Total genomic DNA was extracted from silica gel-dried leaves using a Plant DNA Isolation Kit. Sequencing was performed on an Illumina platform (San Diego, CA), generating paired-end reads. Raw sequencing reads were quality-filtered and adapter-trimmed using fastp v0.23.2 (Chen et al. 2018). The chloroplast genome was assembled from these reads using GetOrganelle v1.7.7.1 (Jin et al. 2020) with default parameters. The assembled genome was annotated using Plastid Genome Annotator (PGA) (Qu et al. 2019) with *B. aromatica* (NC_069835) as the reference, and visualized with CPGview (Liu et al. 2023).

For phylogenetic analysis, the complete chloroplast genome of *B. axillaris* was aligned with those of 13 related species using MAFFT v7.475 (Katoh and Standley 2013), with *Aster ageratoides* (NC_058273) and *Aster tataricus* (NC_042913) designated as outgroups. Poorly aligned regions were removed with Gblocks v0.91b (Castresana 2000). The optimal substitution model (GTR + F + I + G4) was selected by ModelFinder (Kalyaanamoorthy et al. 2017). The maximum-likelihood (ML) phylogenetic tree was inferred using IQ-TREE v2.4.0 (Minh et al. 2020), with 1000 bootstrap replicates. Bayesian’s inference (BI) was performed using MrBayes v.3.2.7 (Ronquist et al. 2012) under the following parameters: two independent runs, each consisting of 100 million generations, with trees sampled every 1000 generations. The resulting tree topology was visualized using iTOL v7.2.2 (https://itol.embl.de) (Letunic and Bork 2024).

## Results

**Figure 2.**
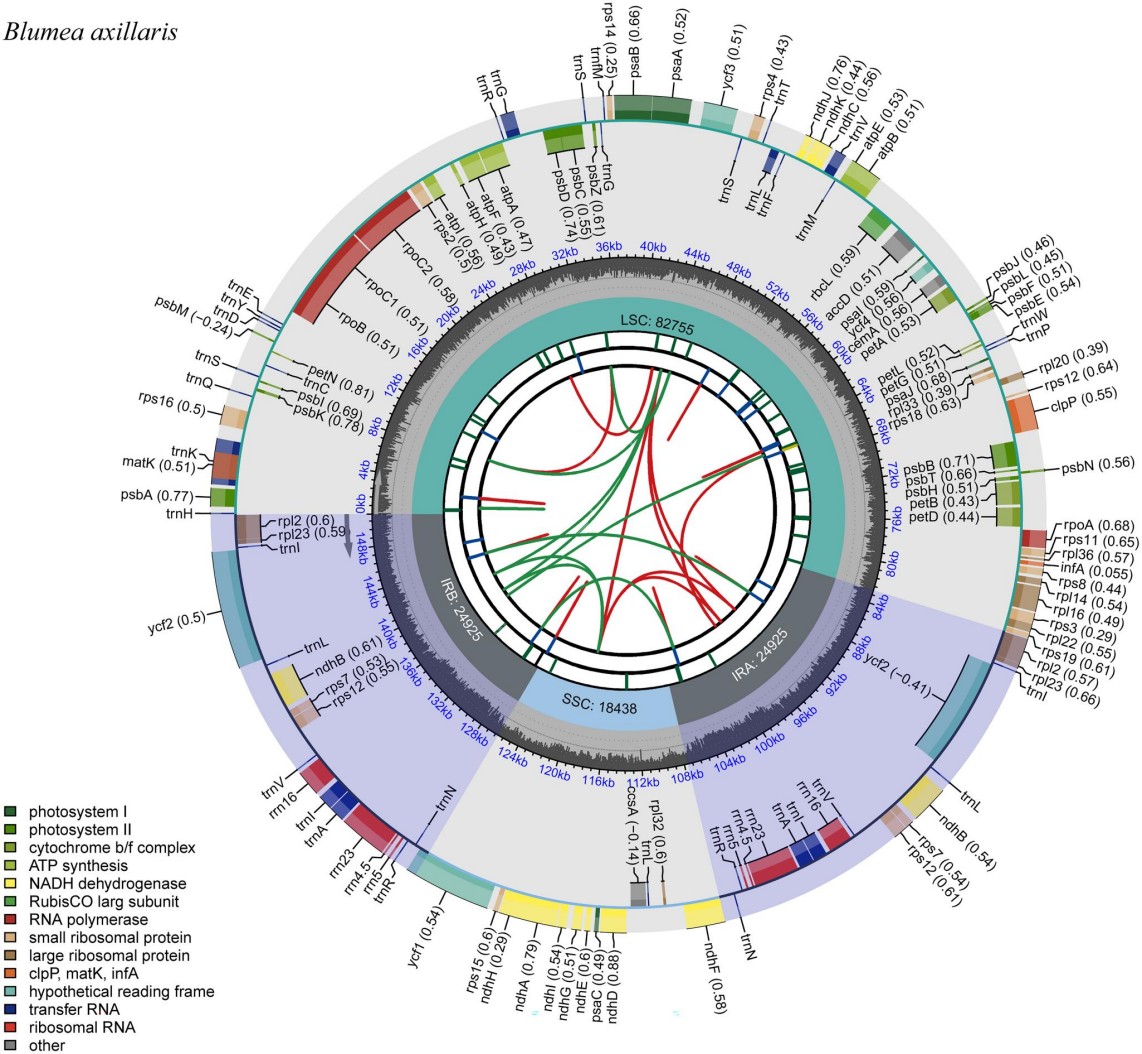
Schematic of the complete chloroplast genome features of *Blumea axillaris*. The species name is labeled at the top-left corner, with six default concentric tracks. From center outward: the innermost track shows dispersed repeats (direct (D) repeats in red arcs, palindromic (P) repeats in green arcs); the 2nd track, long tandem repeats (LTRs) as short blue bars; the 3rd track, short tandem repeats (STRs, microsatellites) as colored short bars for distinction. The 4th track marks key regions: small single-copy (SSC), inverted repeats (IRa, IRb), and large single-copy (LSC) regions. Between the fourth and fifth tracks, the base frequency at each genomic site is displayed; the 5th track shows genome-wide GC content distribution. The outermost track presents gene annotations, with genes on the inner ring transcribed clockwise and those on the outer ring transcribed anticlockwise. A gene functional classification legend is placed at the bottom-left corner.

The assembled genome had an average sequencing depth of 4118.86× (Figure S1). The complete chloroplast genome of *B. axillaris* exhibits a typical quadripartite structure with a total length of 151,043 bp (GenBank accession: PX394520; Figure 2). The overall GC content of the chloroplast genome was 37.49%. The genome consists of an LSC region (82,755 bp), an SSC region (18,438 bp), and two IR regions (24,925 bp each). Compared with the chloroplast genome of *Barnadesia caryophylla* (Barnadesioideae, Asteraceae; OM892817), a large 13 kb inversion containing a nested 4.9 kb small inversion was identified in the LSC region of *B. axillaris* (Figure S2). Annotation revealed 134 functional genes, including 90 protein-coding genes (CDS), 36 tRNA genes, and eight rRNA genes. Among these, 15 genes (*atpF*, *ndhA*, *ndhB*, *petB*, *petD*, *rpl16*, *rpl2*, *rpoC1*, *rps16*, *trnA-UGC*, *trnG-UCC*, *trnI-GAU*, *trnK-UUU*, *trnL-UAA*, and *trnV-UAC)* contained a single intron, while three genes (*clpP1*, *pafI*, and *rps12*) possessed two introns (Figure S3). Additionally, the trans-splicing gene rps12 was present in two copies, each containing three exons and one intron (Figure S4).

**Figure 3.**
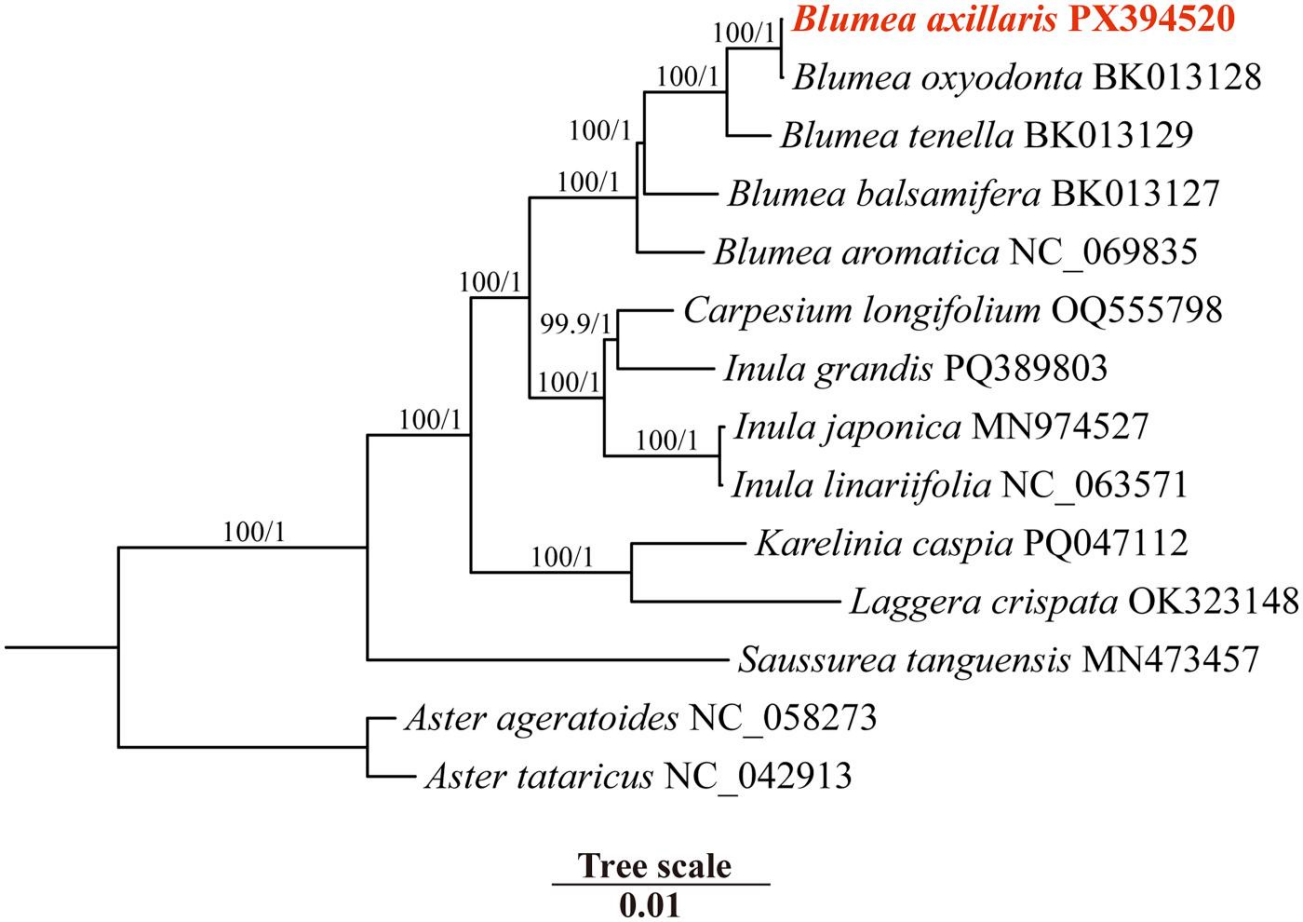
Phylogenetic trees were reconstructed using both maximum-likelihood (ML) and Bayesian inference methods, based on the complete chloroplast genome sequences of *Blumea axillaris* and 13 related taxa. Numbers above the lines represent ML bootstrap values and BI posterior probabilities. Red font indicates the plastome sequence assembled in the current investigation. The 13 species were *Aster ageratoides* (NC_058273, outgroup), *Aster tataricus* (NC_042913, outgroup), *Blumea aromatica* (NC_069835), *Blumea balsamifera* (BK013127) (Abdullah et al. 2021), *Blumea oxyodonta* (BK013128) (Abdullah et al. 2021), *Blumea tenella* (BK013129) (Abdullah et al. 2021), *Carpesium longifolium* (OQ555798) (Chen et al. 2025), *Inula grandis* (PQ389803), *Inula japonica* (MN974527), *Inula linariifolia* (NC_063571), *Karelinia caspia* (PQ047112) (Huang et al. 2025), *Laggera crispata* (OK323148) (Zhou et al. 2022), and *Saussurea tanguensis* (MN473457).

The phylogenetic tree reconstructed from complete chloroplast genomes clearly illustrates the evolutionary relationships among *B. axillaris* and the 13 other Asteraceae species (Figure 3). The topology was well-supported, with all nodes unequivocally resolved (bootstrap values ≥95% for ML and posterior probabilities ≥0.95 for BI). Phylogenetic analysis revealed that *B. axillaris* is most closely related to *B. oxyodonta*. The complete chloroplast genome of *B. axillaris* will enhance our understanding of chloroplast genome evolution and provide evidence for the identification and utilization of *Blumea* species.

## Discussion and conclusions

This study presents the first assembly and annotation of the complete chloroplast genome of *B. axillaris*. The genome is 151,043 bp in length, which is consistent with the sizes reported for other species in the genus *Blumea*. A total of 134 genes were annotated, a number that falls within the typical range for angiosperm chloroplast genomes (110–130 genes) and is slightly higher than that of closely related species such as *B. oxyodonta* (128 genes) (Abdullah et al. 2021). This discrepancy may result from the complete counting of multiple tRNA gene copies or differences in the annotation criteria for certain conserved open reading frames. Besides, structural analysis uncovered a rearrangement in the LSC region that includes one nested inversions, and the double inversion in *B. axillaris* is conserved among most major clades of Asteraceae, with the exception of Barnadesioideae (Asteraceae) (Pascual-Díaz et al. 2021).

Phylogenetic analysis based on the chloroplast genome sequence of *B. axillaris* revealed that *B. axillaris* clusters within a clade containing other species of the genus *Blumea* and related species from other genera. This finding further corroborates the sister-group relationship between *B. axillaris* and *B. oxyodonta* reported previously (Zhang et al. 2019; Chung et al. 2022), with a higher nodal support value. However, due to the limited availability of chloroplast genome data for other *Blumea* species (only four have been reported to date), we could not further confirm whether *B. axillaris* belongs to subbranch II of the *B. lacera* clade. To more fully resolve the taxonomic delimitation and evolutionary history of *B. axillaris* and its close relatives, future studies should integrate comprehensive analyses incorporating broader population sampling (including more species within the genus *Blumea*), nuclear genomic data, more extensive chloroplast genome data, and eco-morphological characteristics.

## Supplementary Material

Supplemental Material

Supplemental Material

Supplemental Material

Supplemental Material

## Data Availability

The sequenced data supporting the findings of this study are openly available in NCBI (https://www.ncbi.nlm.nih.gov) under the accession no. PX394520. The associated BioProject, SRA, and Bio-Sample numbers are PRJNA1344467, SRR35773367, and SAMN52643582, respectively.
