# Supplementary figures and images for: The complete chloroplast genome of Blumea axillaris (Asteraceae: Blumea) and phylogenetic analysis
Source: Mitochondrial DNA B Resour. 2026 Feb 5;11(3):373–7. doi: 10.1080/23802359.2026.2616120 (PMC12880499; doi:10.1080/23802359.2026.2616120)

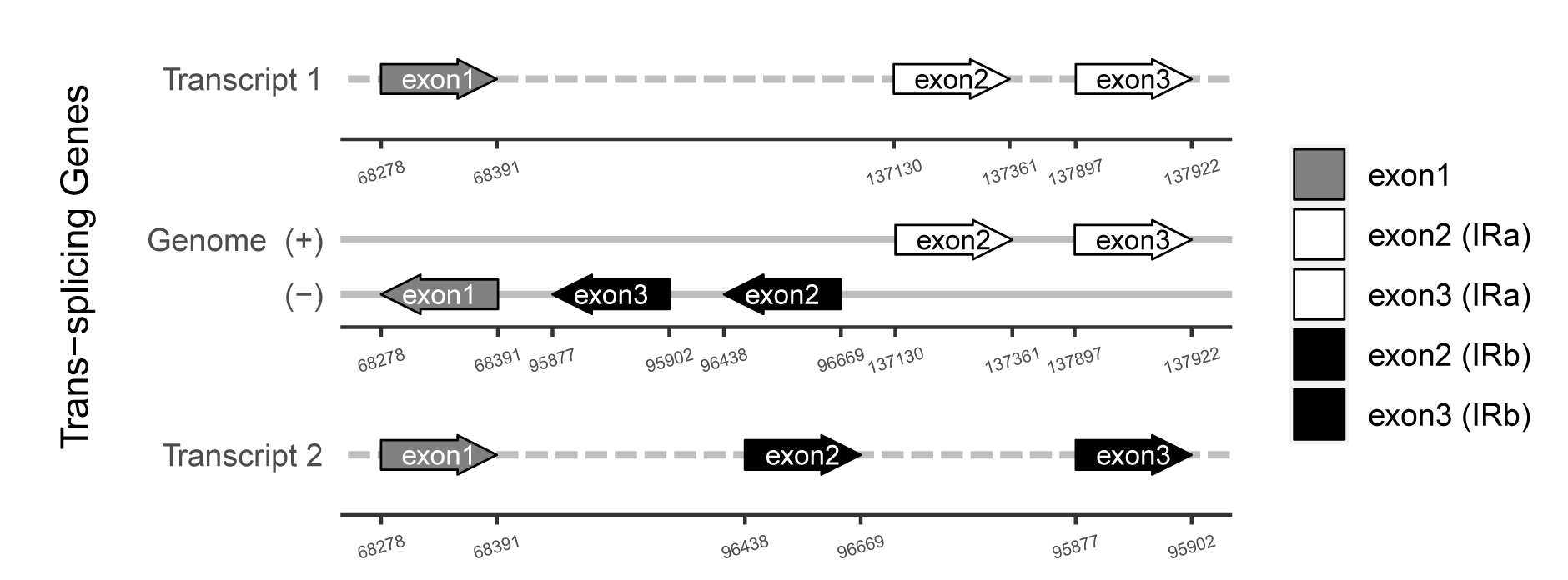

Supplement: Supplemental Material [file TMDN_A_2616120_SM6695.tif]

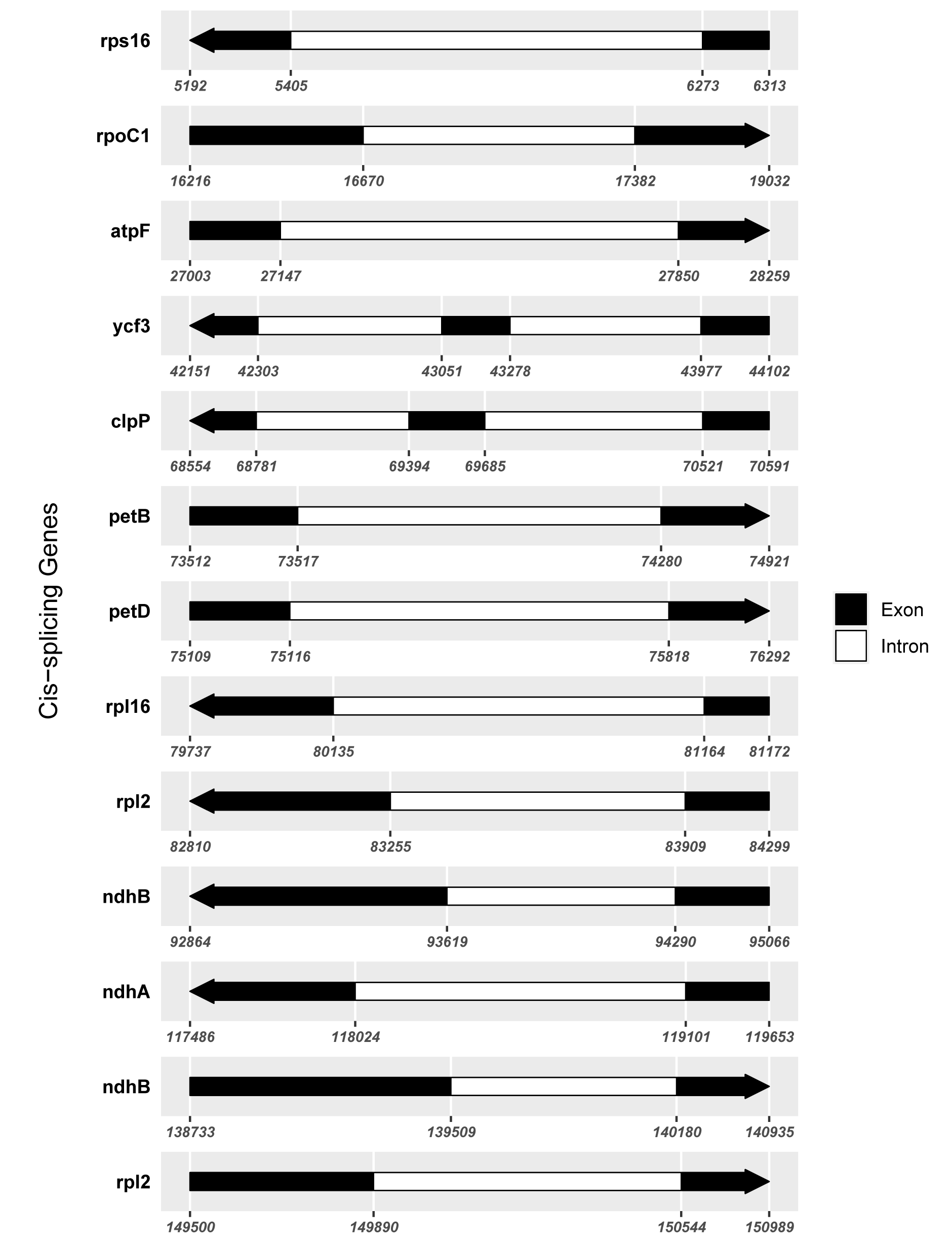

Supplement: Supplemental Material [file TMDN_A_2616120_SM6692.tif]

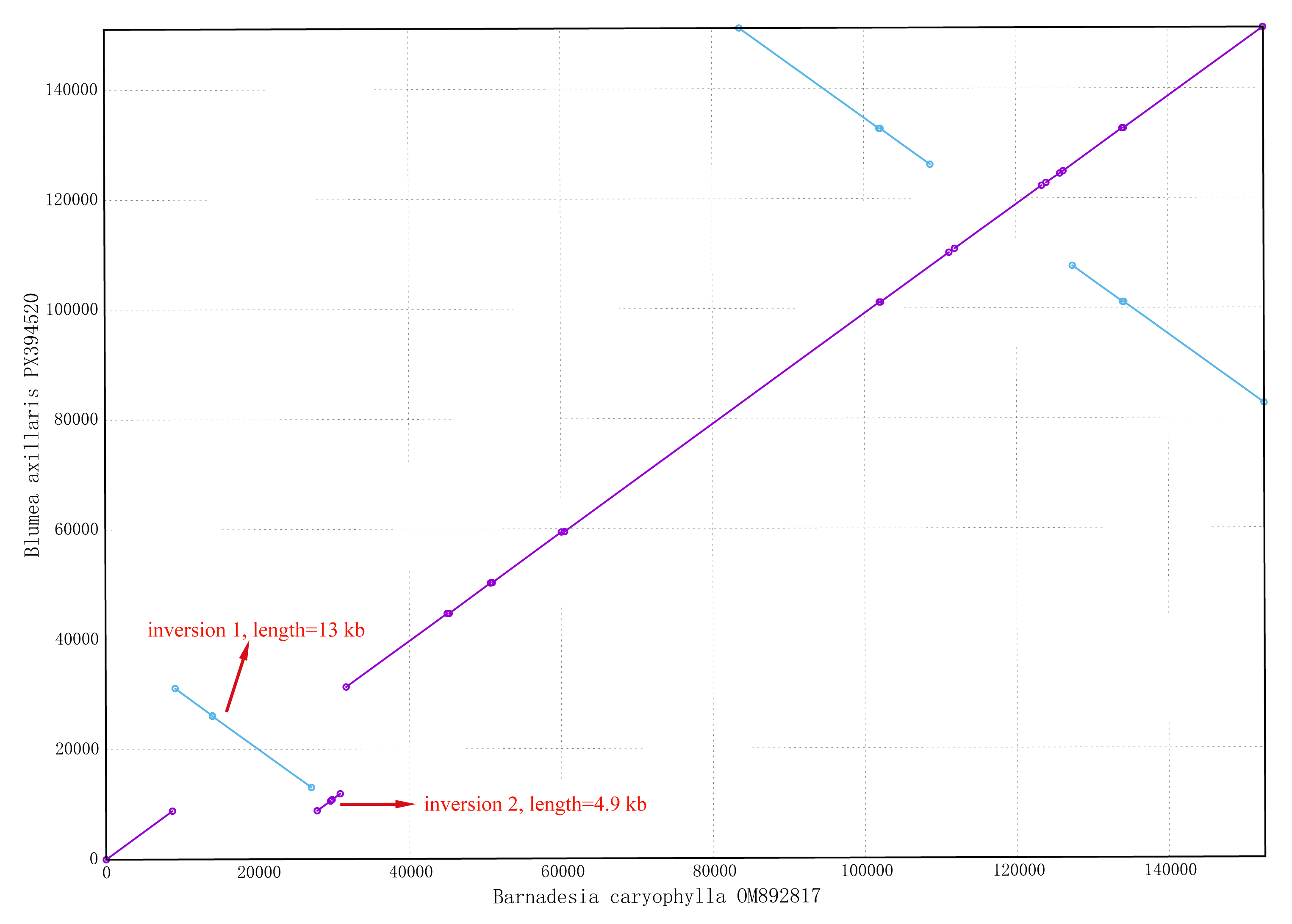

Supplement: Supplemental Material [file TMDN_A_2616120_SM6689.tif]

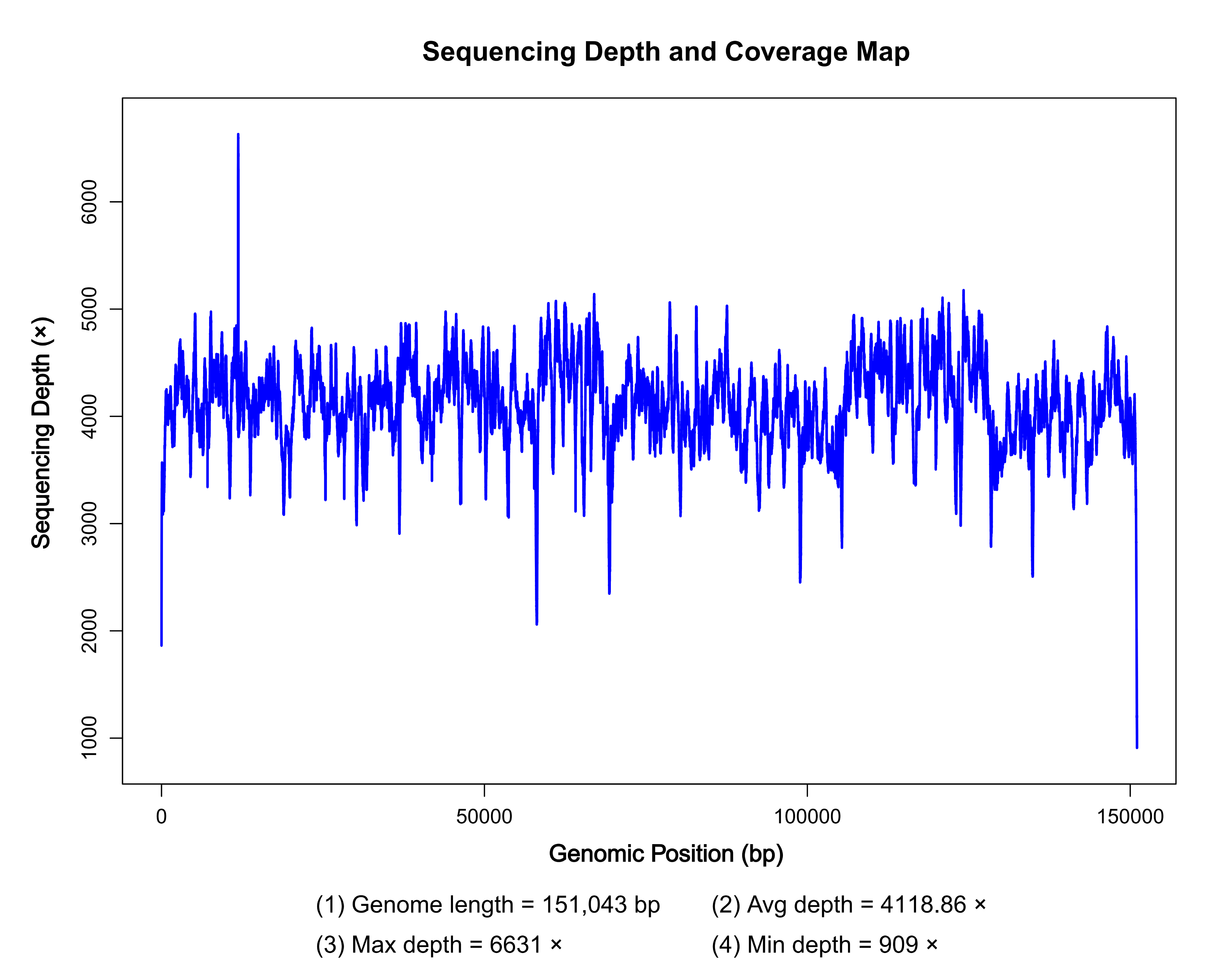

Supplement: Supplemental Material [file TMDN_A_2616120_SM6686.tif]
